# Adiponectin receptors activation performs dual effects on regulating myogenesis and adipogenesis of young and aged muscle satellite cells

**DOI:** 10.1111/cpr.13370

**Published:** 2022-12-09

**Authors:** Shibo Liu, Hanghang Liu, Yao Liu, Ju Zhang, Zhikai Liu, Zizhuo Zheng, En Luo

**Affiliations:** ^1^ State Key Laboratory of Oral Disease, National Clinical Research Center for Oral Diseases West China Hospital of Stomatology, Sichuan University Chengdu China; ^2^ Department of Oral Maxillofacial Surgery West China Hospital of Stomatology, Sichuan University Chengdu China; ^3^ Emergency Department West China Hospital of Stomatology, Sichuan University Chengdu China; ^4^ Maine Medical Center Research Institute Scarborough Maine USA

## Abstract

**Objectives:**

Skeletal muscle mass and function deteriorate with ageing. Adiponectin receptors (APNrs), mainly activated by adiponectin, participate in various physiological activities and have varying signalling pathways at different ages. This study aimed to explore whether discrepant performance exists in APNr activation regulating young and aged muscle satellite cells (MUSCs) and whether age‐related muscle dysfunction could be alleviated upon APNr activation.

**Methods:**

The gastrocnemius muscle phenotype was observed in male mice aged 2 and 18 months. An APNr agonist (AdipoRon) was used *in vitro* and *in vivo* to investigate the changes in cell biological behaviours and whether muscle dysfunction could be retarded after APNr activation.

**Results:**

Aged mice exhibited decreased muscle mass and increased fat infiltration. APNr activation inhibited C2C12 cells and young MUSCs (YMUSCs) proliferation but showed no obvious effect on aged MUSCs (AMUSCs). Moreover, APNr activation inhibited the migration of both YMUSCs and AMUSCs. Interestingly, APNr activation hampered the myogenic differentiation but advanced the adipogenic differentiation of YMUSCs, yet exact opposite results were presented in AMUSCs. It was demonstrated that Wnt and PI3K signalling pathways may mediate the phenotypic differences. Furthermore, *in vivo* experiments verified that APNr activation ameliorated age‐related muscle atrophy and excessive fat infiltration.

**Conclusions:**

APNr activation exerted dual effects on the regulation of myogenesis and adipogenesis of YMUSCs and AMUSCs and rescued age‐related skeletal muscle dysfunction.

## Graphical abstract

Adiponectin receptor (APNr) activation had different effects on myogenic and adipogenic differentiation between aged and young skeletal muscle satellite cells (MUSCs), owing to the different reactions of PI3K and Wnt signalling pathways in young and aged states. Activation of APNr could promote the myogenic differentiation of aged MUSCs and inhibit their adipogenic differentiation, and further improve age‐related skeletal muscle atrophy and reduce fat accumulation.

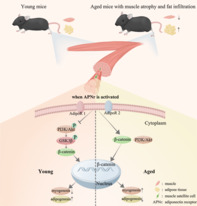

## 1 INTRODUCTION

Skeletal muscle, an important structure and protein reservoir of the body, has certain plasticity and is essential for movement and internal environmental homeostasis including energy storage and glucose metabolism.^1^, ^2^ However, muscle dysfunction, a degeneration in skeletal muscle performance, obviously increases the risk of impaired mobility, falls and fractures and advancing age is undoubtedly one of the most prominent contributors.^3^ One of the hallmark changes is the loss of skeletal muscle mass with functional decline, which is commonly referred to as skeletal muscle atrophy.^4^, ^5^ Moreover, muscles are associated with adipose tissue; the distribution of which changes with age in both physiological and pathological processes. With increasing age, the levels of intramuscular fat increase within and around the fibre bundles, leading to muscle dysfunction.^6^, ^7^ Clinical and preclinical evidence has confirmed that enhancing muscle performance in elderly individuals is achieved by reducing intramuscular lipids content.^8^, ^9^ Pharmacological therapeutic interventions that simultaneously alleviate skeletal muscle atrophy and improve the corresponding lipid metabolism would be an ideal drug therapies for age‐related muscle dysfunction but have not yet been identified.

Skeletal muscle can recover its structural integrity and function in a short time after certain injuries because of its strong regeneration ability. Moreover, the regulation of skeletal muscle maintenance, repair and regeneration is mainly mediated by muscle stem cells, of which muscle satellite cells (MUSCs) play a key role.^4^, ^10^ MUSCs are flat and protuberant cells in skeletal muscle adjacent to the plasma membrane of muscle fibres and lie beneath the basal lamina. MUSCs are generally quiescent and are activated to proliferate and migrate upon injury or external signal intervention.^11^ Proliferating cells finally differentiate into myoblasts and fuse with each other or with existing muscle fibres to form new muscle fibres; this process is regulated by a series of myogenic regulatory factors, including myogenin (MyoG) and MyoD.^12^ Other identified internal or external transcription factors are also involved in regulating muscle regeneration via the interaction of MUSCs with inflammatory cells, stromal cells, nutritional signals and the extracellular matrix.^13^ However, in the aged state, MUSCs lack sufficient regulatory signals, and impaired function weakens their potential for muscle regeneration.^14^ Moreover, ageing is associated with the accumulation of intracellular lipids, and MUSCs have the potential for adipogenic differentiation. Notably, adipocytes and myoblasts are derived from a common pool of progenitor cells that express the early myogenic regulatory factor, Myf5. Genes that monitor myogenesis and adipogenesis are regulated by epigenetic mechanisms, suggesting that either process could be chosen under certain conditions.^15^ Thus, impaired muscle regeneration and increased infiltration of adipose tissue with age cause skeletal muscle dysfunction.

Adiponectin is an important adipocytokine synthesized and secreted by adipocytes. Generally, adiponectin executes multiple metabolic activities, such as improving insulin sensitivity, anti‐inflammatory effects and promoting energy utilization by binding to and activating cell surface receptor‐adiponectin receptors (APNrs), namely AdipoR1 and AdipoR2.^16^, ^17^ As an important target of adiponectin, skeletal muscle can express APNrs, and muscle metabolism is regulated via APNr activation in certain aspects such as muscle regeneration and lipid distribution. The decreased regeneration ability of age‐related muscle stem cells and muscle metabolic disorders could be improved through the adiponectin/AdipoR1 axis‐mediated AMP‐activated protein kinase (AMPK) pathway in senescence‐accelerated mice.^18^ APNr activation via globular adiponectin could inhibit the expression of muscle atrophy marker proteins Atrogin‐1 and muscle ring finger protein‐1 (MuRF‐1), improving muscle atrophy induced by dexamethasone.^19^ In addition, APNr activation can induce goat skeletal MUSCs to differentiate into adipocytes and alleviate the diabetic phenotype.^20^, ^21^ Furthermore, circulating levels and functions of adiponectin differ in multiple physiological and pathological states. However, the mechanism by which APNr activation affects muscle function through downstream signals at different ages remains unknown.

Given the close link between APNr‐mediated signalling and muscle metabolism, we aimed to test the efficacy of APNr activation on MUSC function at different ages. AdipoRon, a newly discovered small molecule activator of APNrs,^22^ was utilized to activate APNrs and explore the effect of APNr on C2C12 cells, young MUSCs (YMUSCs) and aged MUSCs (AMUSCs) to provide a basis for improving muscle metabolism and alleviating age‐related skeletal muscle dysfunction.

## 2 METHODS

### 2.1 Cell culture and reagents

C2C12 cells were cultured in complete Dulbecco's modified Eagle's medium (DMEM) supplemented with 10% foetal bovine serum (FBS; Gibco BRL, Grand Island, New York) in a 37% CO_2_ humidified incubator. Isolation and culture of MUSCs were performed as previously described.^23^ Briefly, the hindlimb muscle tissues were isolated and fully cut into pieces. Then, the pieces were then digested with collagenase I for 1 h and centrifuged at 1500 rpm for 5 min. The supernatant was discarded, and the precipitate was digested with trypsin for 15 min. The pellet was resuspended in a culture flask after centrifugation. The suspension was transferred to a new flask coated with Matrigel for 2.5 h, and AdipoRon (Selleck Chemicals, China) was used to activate APNrs.

### 2.2 Senescence‐associated β‐galactosidase staining

A senescence β‐galactosidase staining kit (Solarbio, Beijing, China) was used to test the expression of senescence‐associated β‐galactosidase in cells from young and aged mice.

### 2.3 Cell proliferation and migration

To test the effect of AdipoRon on C2C12 cells, YMUSCs and AMUSCs, cells were seeded in 96‐well plates at 5 × 10^3^/well for the CCK‐8 assay and in 48‐well plates at 2 × 10^4^/well for the EdU assay. Gradient concentrations of AdipoRon (0, 0.5, 1, 5, 10 and 20 μM) were used to treat the cells. The cells were subjected to a CCK‐8 assay (APExBIO, Houston, Texas) at 24 h and 48 h, according to the manufacturer's protocol. For the EdU cell proliferation assay (iClick EdU Andy Fluor 488 Imaging Kit, GeneCopoeia, USA), the cells were incubated with EdU for 2 h (C2C12 cells) or 3 h (MUSCs) and observed by fluorescence microscopy.

Transwell assays were performed to assess the effect of AdipoRon on MUSC migration. The cells were seeded into the upper chamber at a concentration of 2 × 10^4^/well. Media (containing gradient AdipoRon) with or without 10% serum were added to the lower and upper chambers, respectively. After 18 h, the upper chamber was gently wiped off with cotton swabs, and the cells were fixed with 4% paraformaldehyde and stained with 0.5% crystal violet dye.

### 2.4 Myogenic and adipogenic induction

For myogenic differentiation, the medium was composed of DMEM with 2% (C2C12 cells) or 3% (MUSCs; vol/vol) horse serum (Solarbio, China).

Cells were exposed to induction and differentiation media for adipogenic differentiation. The induction medium contained DMEM, 10% FBS, 0.5 mM IBMX (Sigma‐Aldrich, St. Louis, Missouri), 1.0 μg/ml insulin (Solarbio, China), 1.0 μg/ml rosiglitazone (APExBIO, Houston, Texas) and 0.5 μM dexamethasone (Sigma‐Aldrich); the differentiation medium contained DMEM, 10% FBS, 1.0 μg/mL insulin and 1.0 μg/ml rosiglitazone. Media were alternated every other day between induction and differentiation medium for 8 days, and a differentiation medium was used for the nine‐day maintenance stage. Oil red O staining (Sigma‐Aldrich, St. Louis, Missouri) was performed and observed.

### 2.5 RNA extraction, qRT‐PCR and western blot

Total RNA from cells or muscle tissues was extracted using TRIzol reagent, according to the manufacturer's protocols. RNA was reverse‐transcribed using the PrimeScript™ RT Reagent Kit with gDNA Eraser (Takara, Japan). Quantitative Real‐time Polymerase Chain Reaction (qRT‐PCR) was performed using the SYBR‐Green 2× Master Mix (Bimake, China). The specific primers were listed in Table S1.

For western blotting, cells or muscle tissues were lysed using RIPA lysis buffer (Solarbio, China). Proteins were separated by Sodium dodecyl sulfate polyacrylamide gel electrophoresis (SDS‐PAGE) and transferred to Polyvinylidene fluoride (PVDF) membranes. Primary antibodies and horseradish peroxidase‐labelled secondary antibodies were used for immunoblotting (antibodies are listed in Table S2). An ECL kit was used for visualization.

### 2.6 SiRNA transfection

After cell adherence, AdipoR1 siRNA, AdipoR2 siRNA, AdipoR1 siRNA plus AdipoR2 siRNA and scramble siRNA were prediluted in serum‐free Opti‐MEM medium with 5% EndoFectin (Gene Pharma, China) per well (the siRNA sequences were listed in Table S3). Myogenic and adipogenic induction was performed for another 3 days after 8 h of treatment.

### 2.7 Immunofluorescence

For immunofluorescence, cells were fixed with 4% paraformaldehyde solution for 10 min. After washing three times with phosphate‐buffered saline, the cells were incubated with 0.5% Triton X‐100 for 15 min and blocked with 1% bovine serum albumin for 0.5 h. The primary antibody was added and incubated at 4°C overnight, and the secondary antibody was successively incubated the next day. The cell nuclei were stained with 4',6‐diamidino‐2‐phenylindole (DAPI).

### 2.8 Animal preparation and processing

All animal procedures were approved by the Animal Care Committee of Sichuan University. Male C57BL/6 mice aged 2 months (young mice) and 18 months (aged mice) were both obtained from the West China Medical Laboratory Animal Center of Sichuan University. Gastrocnemius muscles from young and aged mice were isolated and weighed for later analysis. Sixty aged mice (18 months old) were randomly divided into three groups. AdipoRon was dissolved in corn oil and intra‐gastrically administered to (0, 5 and 50 mg/kg body weight [BW]) every alternate day. At 2 and 4 weeks, gastrocnemius muscles were obtained, weighed and then subjected to qRT–PCR, western blot and histological detection.

### 2.9 Histology staining

Gastrocnemius samples were used to prepare paraffin and frozen sections. For paraffin sections, samples were embedded in paraffin, and 4‐μm‐thick sections were acquired. After de‐waxing and rehydration, the sections were subjected to haematoxylin and eosin (HE) staining (Solarbio, China). Frozen sections were also acquired after dehydration, embedded with optimal cutting temperature compound, and subjected to oil red O staining (Solarbio, China). The cross‐sectional area (CSA) and Feret's diameter of the muscle fibres were determined using ImageJ software. For immunofluorescence staining of paraffin sections, antigen retrieval was performed after deparaffinization, and the subsequent procedures were similar to those used for immunofluorescence staining.

### 2.10 Statistical analysis

Quantitative data are presented as the means ± SD. Comparisons between groups were performed using one‐way ANOVA. The IBM SPSS Statistical software (version 20.0, IBM Corp., Armonk, New York) was used to analyse the experimental results. The significant *p*‐value was set to <0.05.

## 3 RESULTS

### 3.1 Skeletal muscle atrophy and intermuscular adipose tissue infiltration appeared in aged mice

**FIGURE 1:**
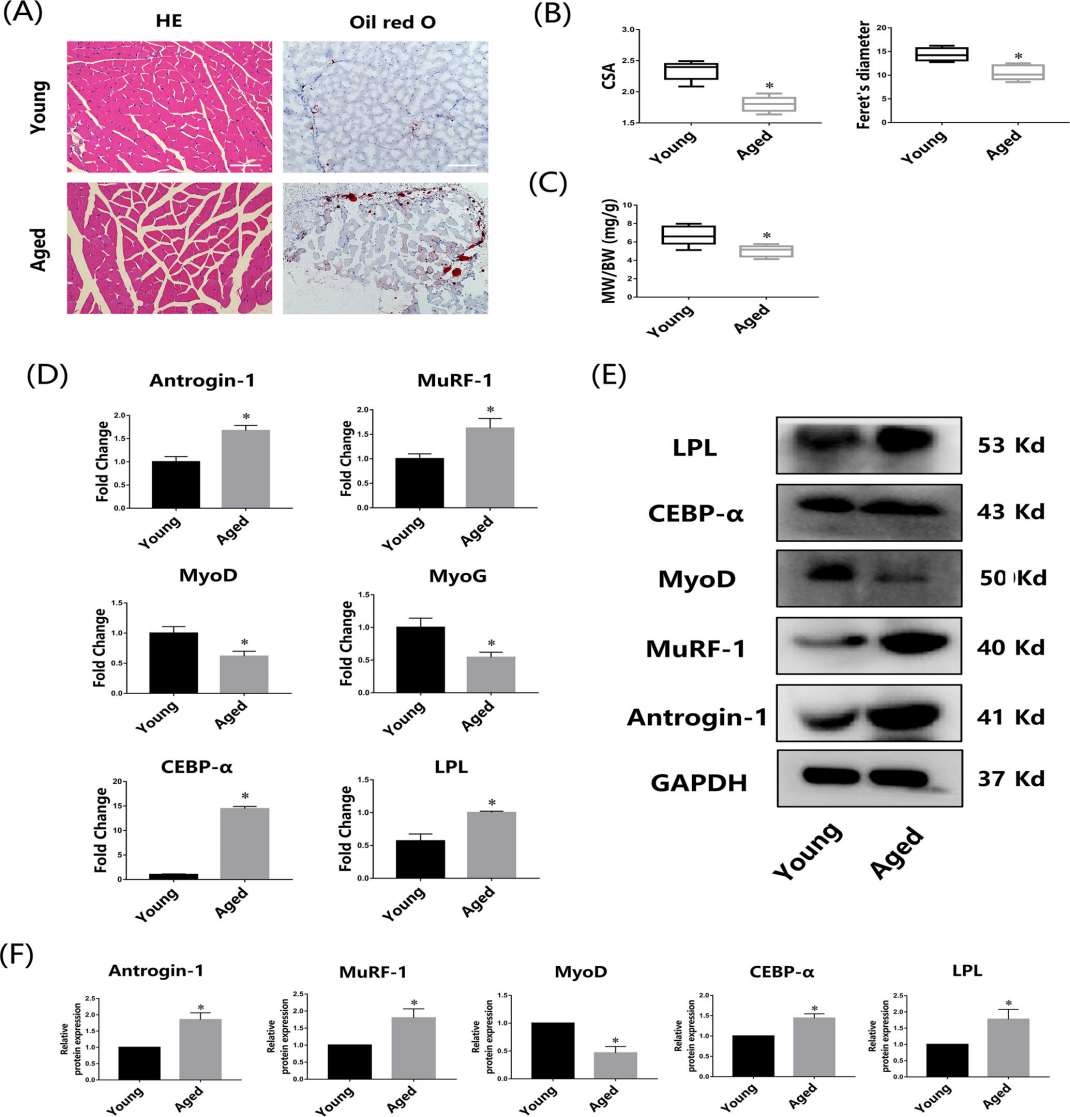
Skeletal muscle atrophy and intermuscular adipose tissue infiltration were discovered in aged mice. (A) Haematoxylin and eosin (HE) and oil red O staining of gastrocnemius muscles from young and aged mice. Bar =100 μm. (B), (C) Cross‐sectional area (CSA), Ferret's diameter, and muscle weight/body weight ratio of muscles in young and aged mice. (D), (E) Gene and protein expression in gastrocnemius muscles isolated from young and aged mice. (F) Quantitative analysis of protein expression levels between young and aged mice. *p < 0.05, compared with the young group. Data are presented as the mean ± SD (n = 10 male C57BL/6 mice/group).

Phenotypic characteristics of skeletal muscle from 2‐month‐old and 18‐month‐old mice were evaluated. HE staining indicated that both CSA and Feret's diameter of the muscle fibres were reduced in aged mice compared with young mice, and aged mice also showed an increase in fat mass according to the oil red O staining (Figure 1A,B). In addition, muscle weight relative to BW (MW/BW) significantly decreased with age (Figure 1C). The results of qRT‐PCR showed that Atrogin‐1 and MuRF‐1, two representative proteins of muscle atrophy, were highly expressed in aged muscle samples (Figure 1D). Moreover, the results also suggested that myogenic factors (MyoD, MyoG) declined with age, while higher expression of adipogenic factors (CEBP‐α and LPL) was observed in aged mice (Figure 1D). Consistently, results of western blot and the subsequent quantitative analysis further confirmed these findings (Figure 1E,F). In addition, a greater amount of inguinal fat was observed in aged mice compare with young mice (Figure S1).

### 3.2 AMUSCs showed a senescent phenotype and impaired myogenesis compared with YMUSCs

**FIGURE 2:**
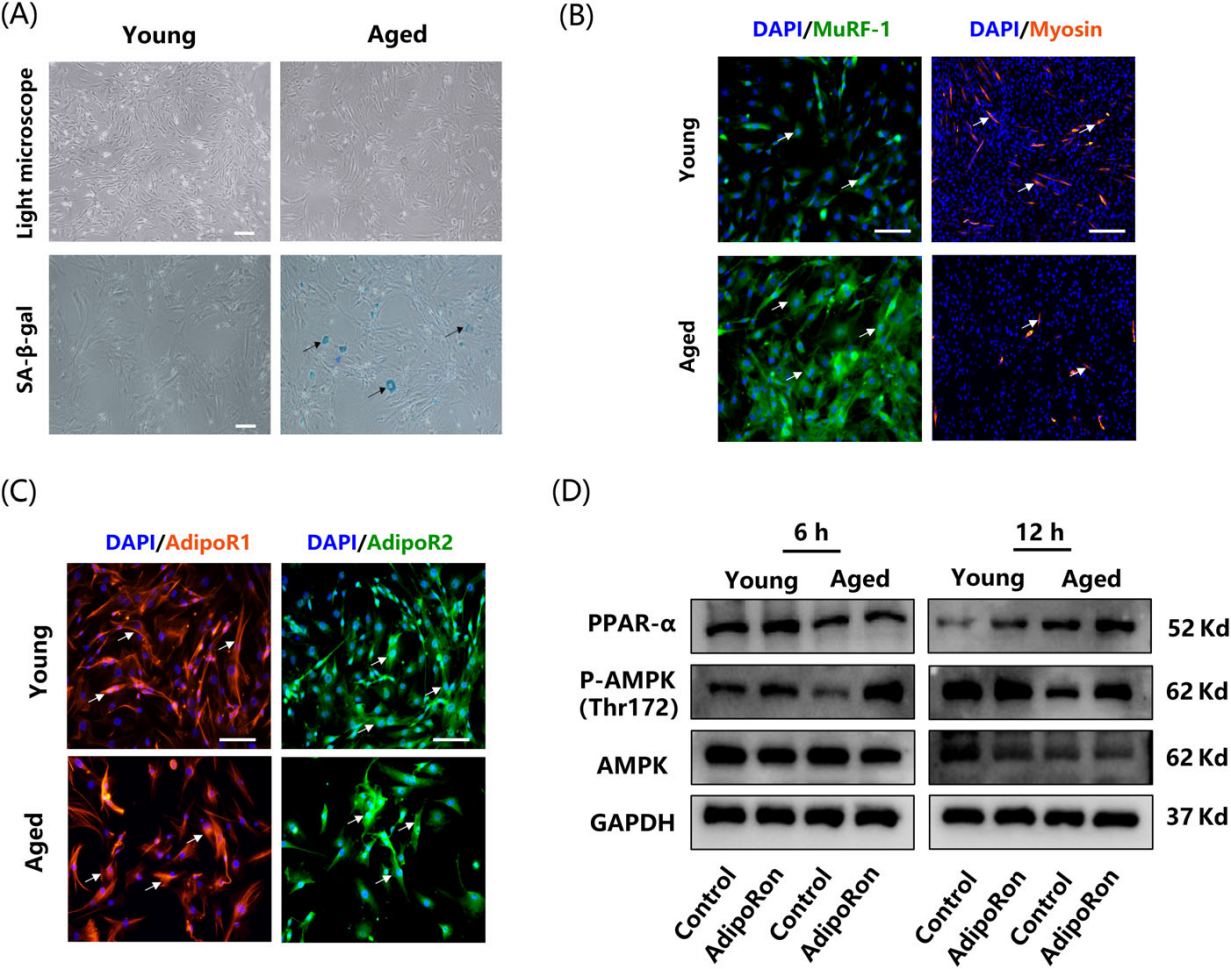
Morphology and characterization of muscle satellite cells (MUSCs). (A) Light microscopy photographs and SA‐β‐galactosidase (SA‐β‐gal) staining of MUSCs from young and aged mice. Bar =200 μm. (B) Immunofluorescence staining of MuRF‐1 (white arrows on the left) and myosin (white arrows on the right) in MUSCs from young and aged mice. Bar =100 μm. (C) AdipoR1 (white arrows on the left) and AdipoR2 (white arrows on the right) staining of MUSCs from young and aged mice. Bar =100 μm. (D) Western blot results of changes in the AMPK and PPAR‐α signalling pathways following AdipoRon treatment in YMUSCs and AMUSCs. Data are presented from at least three independent experiments.

YMUSCs and AMUSCs were isolated from young and aged mice, respectively. SA‐β‐gal staining showed that more senescent cells presenting blue staining were found in AMUSCs (Figure 2A). In addition, immunofluorescence detection verified that MuRF‐1 was highly expressed in AMUSCs compare with YMUSCs, and less myosin staining in AMUSCs hinted its impaired capability to form myotubes (Figure 2B). Furthermore, the expression of AdipoR1 and AdipoR2 was observed in both YMUSCs and AMUSCs and the results of immunofluorescence staining showed that they were both distributed in the cytoplasm and cytomembrane (Figure 2C). Activation of AMPK and peroxisome proliferator‐activated receptor‐α (PPAR‐α) signalling pathways upon AdipoRon treatment were confirmed by western blot, suggesting that APNr activation was achieved in both YMUSCs and AMUSCs (Figure 2D).

### 3.3 APNr activation inhibited proliferation and myogenesis but promoted adipogenesis of C2C12 cells

**FIGURE 3:**
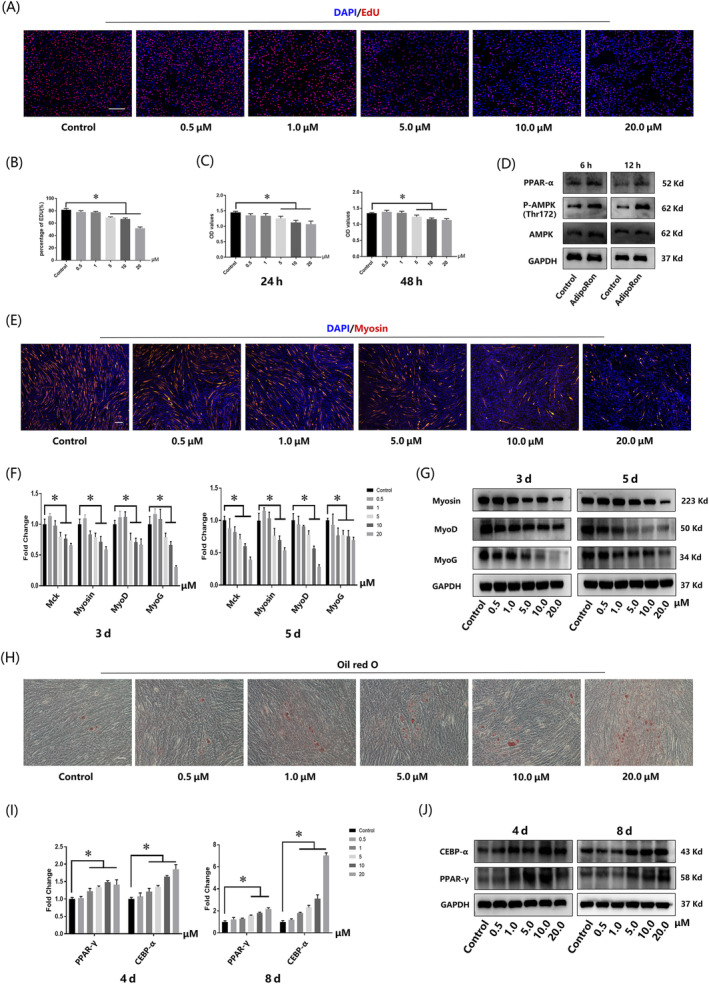
Adiponectin receptor activation inhibited proliferation and myogenesis but promoted adipogenesis of C2C12 cells. (A), (B) EdU staining and quantification analysis of C2C12 cells after 24 h AdipoRon treatment. Bar =100 μm. (C) CCK‐8 assay of C2C12 cells after 24 and 48 h AdipoRon treatment. (D**)** Western blot results of changes in the AMPK and PPAR‐α signalling pathways in C2C12 cells. (E) Myosin staining of C2C12 cells after 5 days of myogenic differentiation. Bar =200 μm. (F), (G) qRT‐PCR and western blot results of C2C12 cells after 3 and 5 days of myogenic differentiation. (H) Representative photographs of oil red O staining after 17 days of adipogenic differentiation and AdipoRon treatment. Bar =200 μm. (I), (J) qRT‐PCR and western blot results of C2C12 cells after 4 and 8 days of adipogenic differentiation and AdipoRon treatment. *p < 0.05, compared with the control group. Data are presented as the mean ± SD from at least three independent experiments.

EdU analysis (Figure 3A,B) and CCK‐8 assay (Figure 3C) indicated that AdipoRon (>5 μM) slightly inhibited the proliferation of C2C12 cells. APNr activation upon AdipoRon treatment was further testified via activated AMPK and PPAR‐α signalling pathways (Figure 3D). The effect of APNr activation on C2C12 cell differentiation was then explored. Immunofluorescence staining after 5 days of myogenic induction showed that myogenesis of C2C12 cells was suppressed in a concentration‐dependent manner (Figure 3E). The qRT‐PCR and western blot assays showed that the expression of myogenic markers, such as muscle creatine kinase (Mck), MyoG, MyoD and myosin. was also downregulated after AdipoRon treatment (Figure 3F,G). Moreover, C2C12 cells showed an opposite trend of adipogenesis after treatment with gradient concentrations of AdipoRon. Furthermore, the adipogenic markers CEBP‐α, PPAR‐γ and LPL were enhanced at the gene and protein levels, and oil red O staining showed a consistent result (Figures 3H–J and S2A).

### 3.4 APNr activation inhibited the proliferation and migration ability of YMUSCs but only inhibited the migration of AMUSCs

**FIGURE 4:**
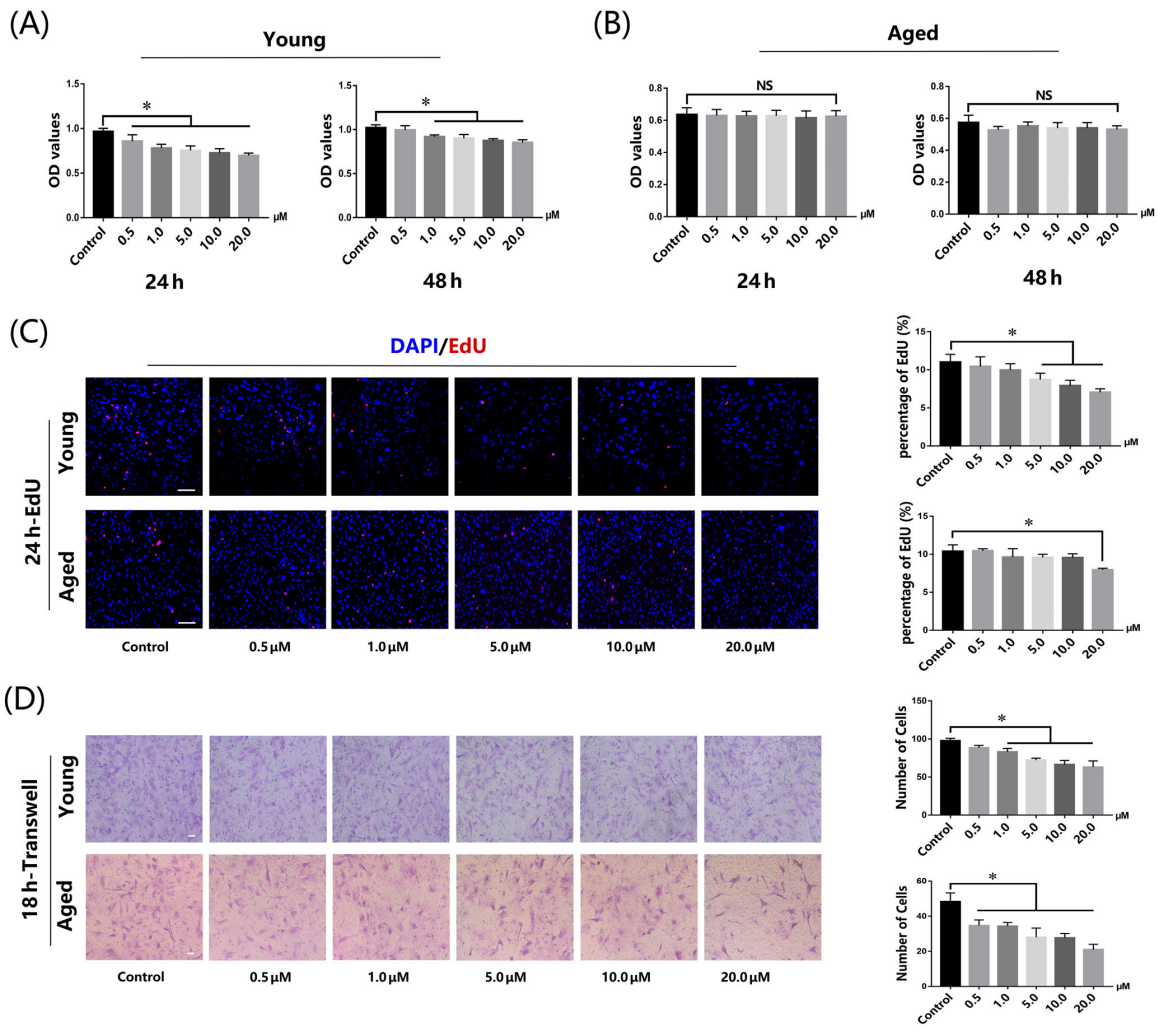
Adiponectin receptor activation slightly inhibited young muscle satellite cell (YMUSC) and aged muscle satellite cell (AMUSC) proliferation and migration, but showed no obvious effect on AMUSC proliferation. (A,B) CCK‐8 assays of YMUSCs and AMUSCs after 24 and 48 h AdipoRon treatment. (C) Representative images and quantification analysis for EdU staining of YMUSCs and AMUSCs after 24 h AdipoRon treatment. Bar =100 μm. (D) Representative images and quantification analysis of Transwell assays of YMUSCs and AMUSCs after 18 h AdipoRon treatment. Bar =200 μm. NS: *p* > 0.05; *p < 0.05, compared with the young group. Data are presented as the mean ± SD from at least three independent experiments.

CCK‐8 and EdU assays suggested that AdipoRon treatment reduced the proliferation ability of YMUSCs, but no significant change was found in AMUSCs—little inhibitory effect was observed at 20 μM for AdipoRon treatment in the EdU assay (Figure 4A–C). In addition, the inhibitory effect of APNr activation on the migration activity of YMUSCs and AMUSCs were both positively correlated with the AdipoRon concentration (Figure 4D).

### 3.5 APNr activation presented dual effects on myogenesis and adipogenesis of YMUSCs and AMUSCs

**FIGURE 5:**
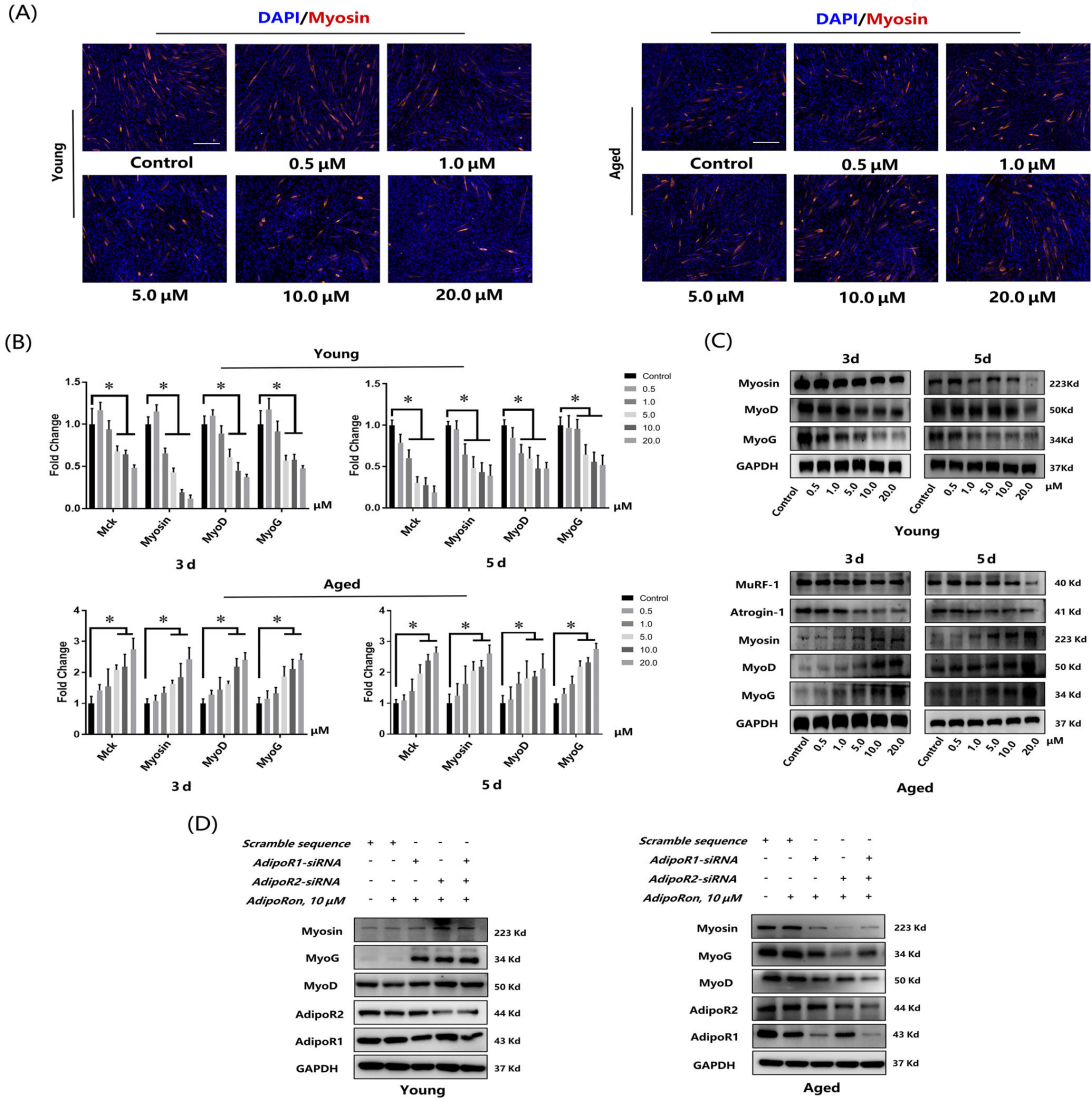
Adiponectin receptor activation displayed opposite effects on the myogenesis of young muscle satellite cells (YMUSCs) and aged muscle satellite cells (AMUSCs). (A) Myosin staining of YMUSCs and AMUSCs after 5 days of myogenic differentiation. Bar =100 μm. (B) qRT‐PCR results of YMUSCs after 3 and 5 days of myogenic differentiation. (C) Western blot results of AMUSCs after 3 and 5 days of myogenic differentiation. (D) Western blot results of YMUSC and AMUSC myogenesis ability after AdipoR1 and AdipoR2 knockdown. *p < 0.05, compared with the young group. Data are presented as the mean ± SD from at least three independent experiments.

Immunofluorescence staining of myosin indicated that YMUSCs reacted to APNr activation similarly to C2C12 cells: myotube formation was repressed by AdipoRon in a concentration‐dependent manner, while a significantly advanced effect on myotube formation was found in AMUSCs (Figure 5A). The qRT‐PCR and western blot assays showed the consistent tendency: expression of myogenic factors (Mck, myosin, MyoD and MyoG) was inhibited in YMUSCs but promoted in AMUSCs upon AdipoRon treatment (Figure 5B,C). In addition, muscle atrophy markers (Atrogin‐1 and MuRF‐1) were inhibited by AdipoRon treatment in a dose‐dependent manner in AMUSCs (Figure 5C). Additionally, knockdown of AdipoR1 or AdipoR2 could abrogate the inhibitory effect in YMUSCs and the promoted effect in AMUSCs upon AdipoRon treatment (Figure 5D).

**FIGURE 6:**
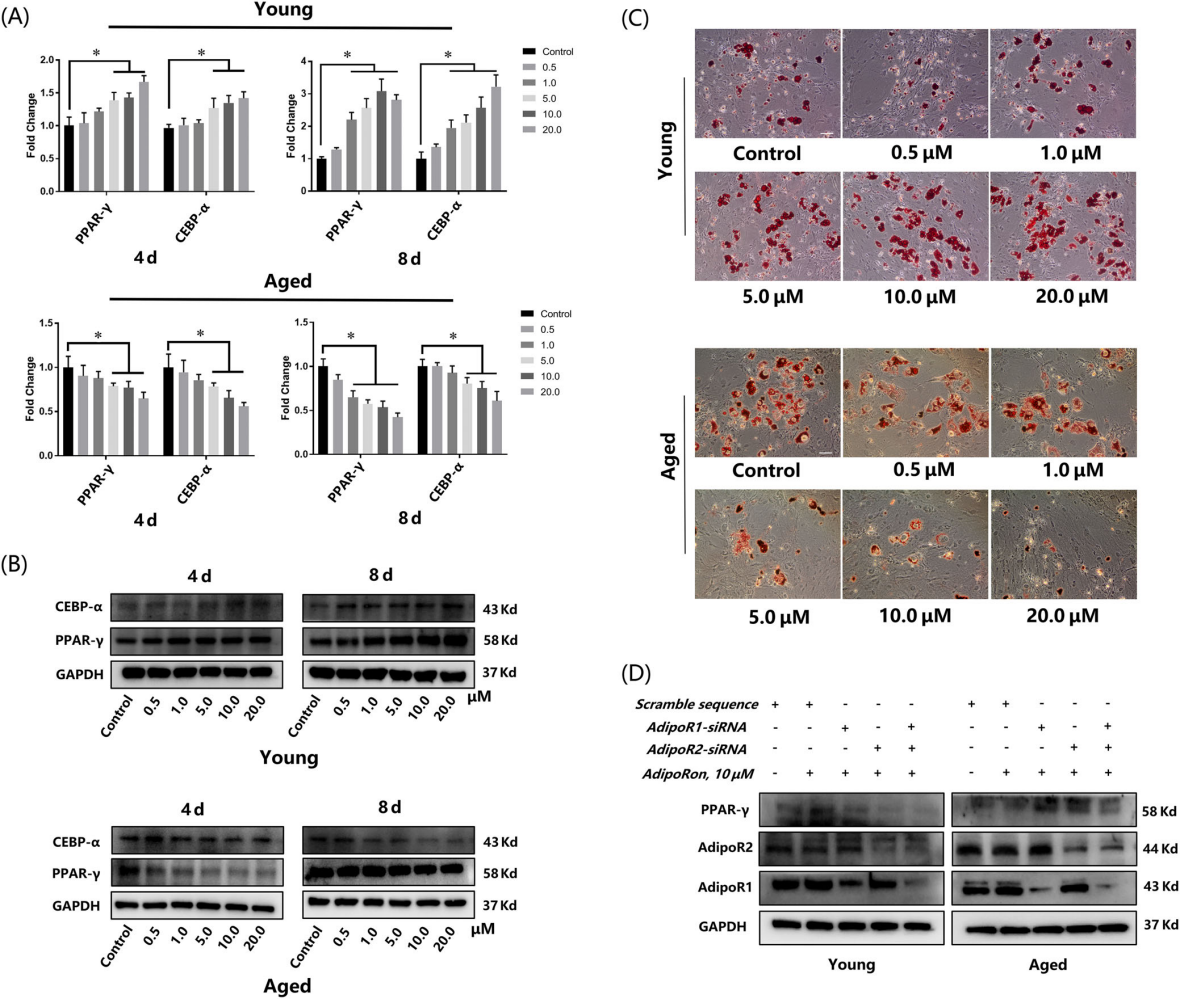
Adiponectin receptor activation displayed opposite effects on the adipogenesis of young muscle satellite cells (YMUSCs) and aged muscle satellite cells (AMUSCs). (A) qRT‐PCR results of YMUSCs and AMUSCs after 4 and 8 days of adipogenic differentiation. (B) Western blot results of YMUSCs and AMUSCs after 4 and 8 days of adipogenic differentiation. (C) Oil red O staining of YMUSCs and AMUSCs after 17 days of adipogenic differentiation. Bar =200 μm. (D) Western blot results of the adipogenesis ability of YMUSCs and AMUSCs after AdipoR1 and AdipoR2 knockdown. *p < 0.05, compared with young group. Data are presented as the mean ± SD from at least three independent experiments.

APNr activation had opposite effects on the adipogenesis of YMUSCs and AMUSCs. Similarly, qRT‐PCR, western blot and oil red O staining also showed that advanced lipid droplet formation and the expression of adipogenic genes and proteins were observed in YMUSCs, whereas impaired adipogenesis was demonstrated in AMUSCs (Figures 6A–C and S2B,C). Likewise, the effects of AdipoRon on YMUSC and AMUSC differentiation were significantly reversed after AdipoR1 and AdipoR2 knockdown (Figure 6D).

### 3.6 Wnt and PI3K signalling pathways may mediate the phenotypic differences between YMUSCs and AMUSCs upon APNr activation

**FIGURE 7:**
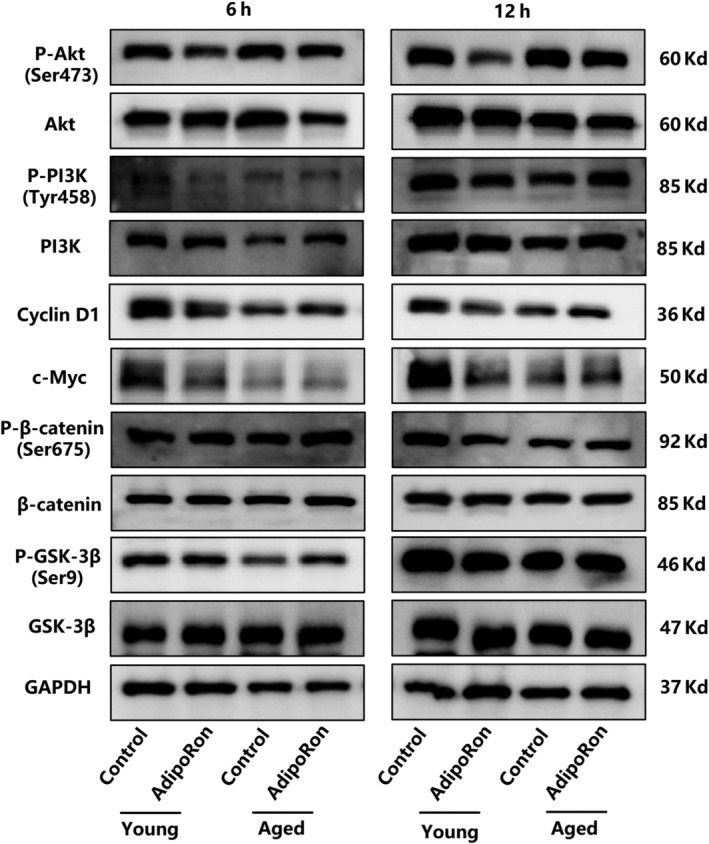
Adiponectin receptor activation showed different regulation of the Wnt and PI3K signalling pathways in young muscle satellite cells and aged muscle satellite cells. Data are presented from at least three independent experiments.

For YMUSCs, the expression of phosphorylated PI3K, AKT, GSK3β, β‐catenin (phosphorylation at Ser675 which promotes β‐catenin nuclear translocation), c‐Myc and cyclin D1 (downstream factors of the Wnt signalling pathway) were inhibited (Figure 7). However, no significant changes in the PI3K signalling pathway were observed in AMUSCs after AdipoRon treatment. Conversely, the Wnt signalling pathway was activated in AMUSCs after 6 and 12 h of AdipoRon treatment, as evidenced by increased expression of phosphorylated β‐catenin (Ser675), c‐Myc and cyclin D1 (Figure 7).

### 3.7 APNr activation improved age‐related skeletal muscle dysfunction in aged mice

**FIGURE 8:**
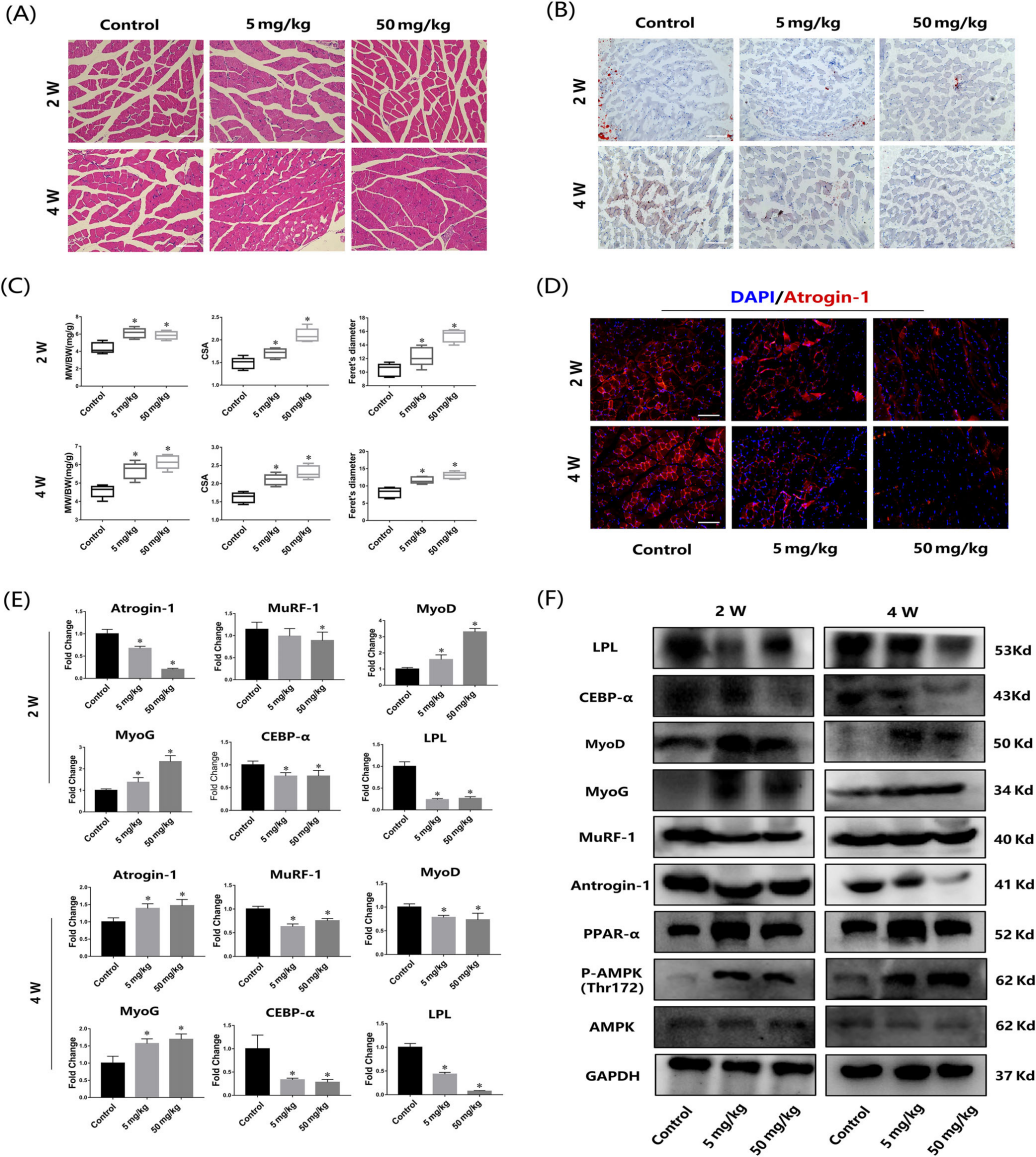
Adiponectin receptor activation suppressed fat infiltration and rescued age‐induced skeletal muscle dysfunction. (A) Representative HE staining of skeletal muscle after AdipoRon treatment for 2 and 4 weeks. Bar =100 μm. (B) Fat infiltration of skeletal muscles upon treatment with different concentrations of AdipoRon treatment for 2 and 4 weeks. Bar =100 μm. (C) Muscle weight/body weight ratio, cross‐sectional area and Ferret's diameter measured from muscle samples of aged mice after AdipoRon treatment for 2 and 4 weeks. (D) Antrogin‐1 immunofluorescence staining of skeletal muscle after AdipoRon treatment for 2 and 4 weeks. Bar =100 μm. (E,F) qRT‐PCR and western blot results of muscles after 2 and 4 weeks of AdipoRon treatment. *p < 0.05, compared with the young group. Data are presented as the mean ± SD (*n* = 10 male C57BL/6 mice/group).

The gastrocnemius muscles were obtained after 2 and 4 weeks of AdipoRon treatment. HE staining indicated that CSA and Feret's diameter of muscle fibres in both AdipoRon treatment groups increased compared with the control group (Figure 8A,C), and the statistical results of MW/BW showed the same tendency (Figure 8C). Oil red O staining showed that fat infiltration was alleviated by AdipoRon treatment (Figure 8B). Moreover, AdipoRon administration caused no significant change in BW (Figure S3A). In addition, the results of immunofluorescence staining, qRT‐PCR and western blot showed that AdipoRon inhibited the expression of muscle atrophy markers (Atrogin‐1 and MuRF‐1) and adipogenic markers (CEBP‐α and LPL) and promoted the expression of myogenic markers (MyoD and MyoG) as the concentration increased (Figure 8D–F). Moreover, the AMPK and PPAR‐α signalling pathways were both activated after AdipoRon treatment (Figure 8F). Furthermore, inguinal fat was reduced after the administration of AdipoRon, and no significant drug toxicity‐related injuries were found in the heart, kidney or liver samples (Figure S3B,C).

## 4 DISCUSSION

**FIGURE 9:**
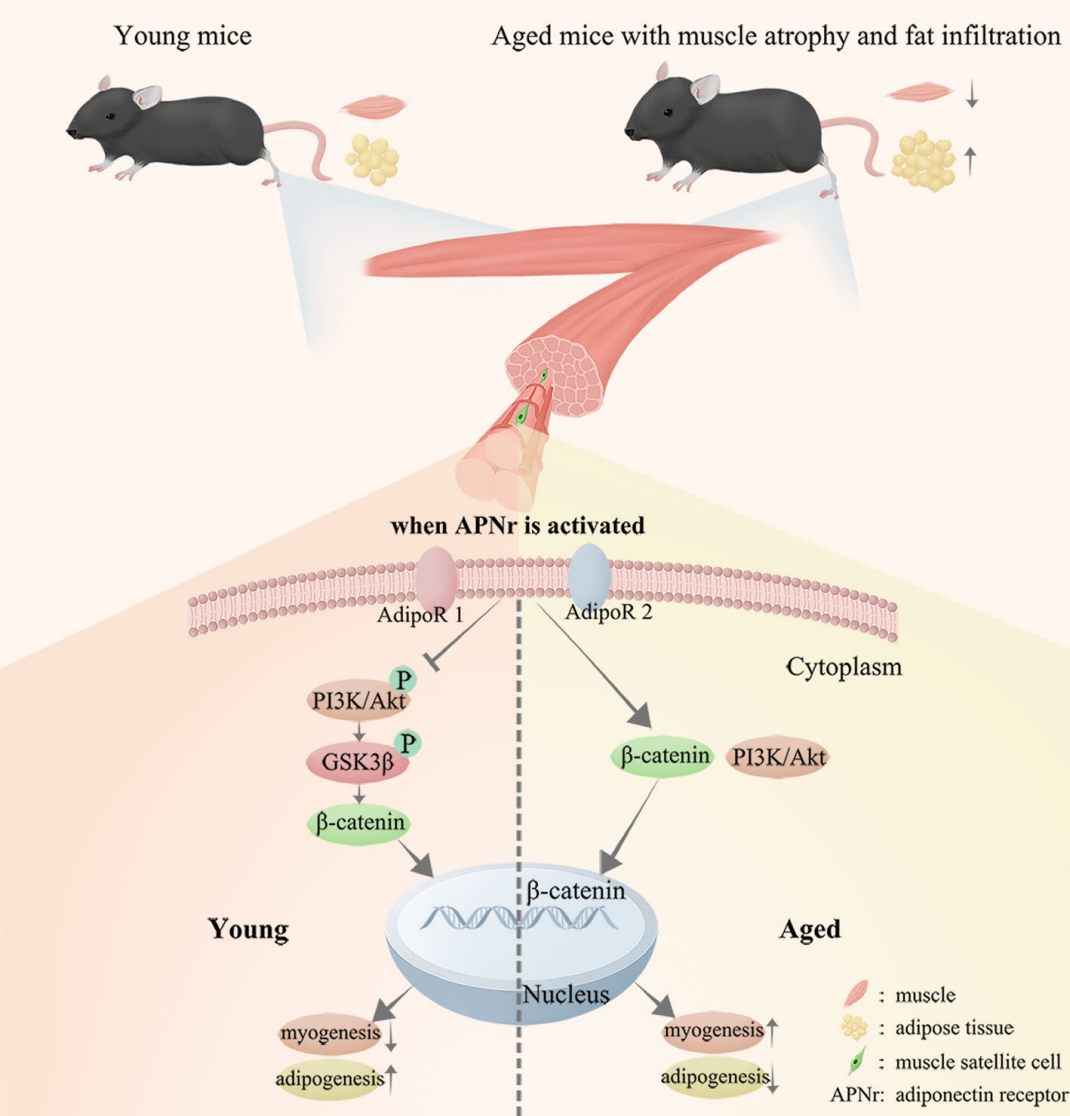
A sketch map of the dual effects of adiponectin receptor (APNr) activation on regulating muscle satellite cell function at different ages.

Skeletal muscle dysfunction is an age‐induced decline in muscle strength, accompanied by a loss of muscle mass and accumulation of adipose tissue.^24^, ^25^ In this study, we used AdipoRon, an APNr agonist, to explore the effect of APNr activation on skeletal muscle function. Interestingly, opposite responses were observed in young and aged MUSCs upon APNr activation. AdipoRon impaired the myogenic capability of C2C12 cells and YMUSCs but promoted adipogenesis, whereas AMUSCs showed enhanced myogenesis and inhibited adipogenesis after APNr activation. Different changes in the Wnt and PI3K signalling pathways after APNr activation in the young and aged states may shed light on the aforementioned effects (Figure 9). Moreover, in vivo experiments revealed that APNr activation improves age‐related skeletal muscle atrophy and suppresses fat infiltration.

Since its discovery, the effects of adiponectin on diverse metabolic activities mediated by APNr activation have been widely studied, including bone, lipid and muscle metabolism.^26^, ^27^, ^28^ Adiponectin is a systemic bioactive hormone that exerts physiological functions by binding to APNrs (AdipoR1 and AdipoR2). AdipoR1 and AdipoR2 are both abundantly expressed in skeletal muscle and share certain similarities in sequence and ligand‐binding sites.^29^ They are predicted to have seven transmembrane domains but are distinct from G protein‐coupled receptors.^30^ AdipoR1 activates the AMPK pathway, and AdipoR2 activates the PPAR‐α pathway; adiponectin binds to the two receptors and regulates glucose uptake, lipid oxidation and other metabolic processes.^31^, ^32^ The specific knockout of muscle AdipoR1 leads to a decrease in AMPK activity and involuntary motor ability.^33^ Adiponectin intervention in C2C12 cells induced AMPK phosphorylation and PGC1‐α activation, which required AdipoR1 signal transduction.^34^ However, the large size and complex polymerization characteristics of adiponectin make its extensive use inconvenient, and alternative APNr agonists have been explored in APNr activation. AdipoRon is a small molecule compound that can act precisely on APNrs. As an effective substitute for adiponectin, it was applied in this research to act on APNrs (AdipoR1 and AdipoR2). Similar to previous studies, our study showed that regardless of age, the AMPK and PPAR‐α signalling pathways were activated after AdipoRon treatment, suggesting that APNr activation was achieved.

MUSCs have been shown to possess myogenic and adipogenic abilities.^35^, ^36^ Numerous studies have shown that the function of MUSCs is impaired with age.^37^, ^38^ As part of the ageing process, MUSCs suffer from impaired myogenic ability and decreased regulation of the cell cycle and cell fate, which causes adipose tissue accumulation, leading to muscle dysfunction finally occurs.^39^ Consistent with previous predictions, skeletal muscle atrophy and adipose tissue accumulation were observed in aged mice in this study. Moreover, we investigated the combined effects of APNr activation on the myogenic and adipogenic activities of MUSCs for the first time. Overall, cell proliferation and migration were slightly inhibited after APNr activation, but the opposite differentiated behaviours were observed in the young and aged states. These data suggest that the effects of APNr activation on MUSC function may be related to age, a prospect that deserves further exploration.

A broader investigation aimed downstream of the adiponectin signalling pathway is also needed to demonstrate further mechanisms that clarify age‐related phenotypic differences. In this study, we found that the different manifestations of MUSC differentiation in young and aged states had little to do with the AMPK and PPAR‐α signalling pathways; in contrast, the Wnt and PI3K signalling pathways play an important role in adiponectin signalling pathways and are both involved in modulating muscle and adipose metabolism.^40^, ^41^ The activated PI3K signalling pathway can attenuate lipid accumulation, rescues myotube formation and ameliorate skeletal muscle atrophy.^42^, ^43^, ^44^, ^45^ Wnt signalling participates in the regulation of satellite cell differentiation and self‐renewal, and this pathway is poorly activated in mature skeletal muscles.^46^ Activated β‐catenin signalling can increase myogenin expression to stimulate myoblast differentiation and inhibit adipogenic differentiation.^47^ Stabilized β‐catenin can govern skeletal muscle fibro/adipogenic progenitor adipogenesis and repress PPAR‐γ expression.^41^ Moreover, the two signalling pathways are closely connected, and PI3K pathway activation can cause a series of changes in the Wnt pathway.^48^ Interestingly, we found that the PI3K/Wnt signalling pathway was inhibited in YMUSCs; in AMUSCs, however, no obvious change was observed in the PI3K pathway, but Wnt pathway activation was evident. Based on the above findings, we inferred that the ageing may disturb the response of the PI3K/Wnt pathway to APNr activation. Although there was no activation of the PI3K pathway, other possible factors could have activated of the Wnt pathway. Studies have verified that changes in sirtuins and mTOR due to ageing may cause changes in the regulatory function of signalling pathways.^49^, ^50^ Furthermore, studies have shown that weakening age‐induced ROS levels can decrease the binding of FoxO to β‐catenin, transforming FoxO‐mediated transcription into original T‐cell factor (TCF)‐mediated transcription and inhibiting the Wnt pathway.^51^ Certainly, the specific factors causing these differences require further exploration.

The activation of APNrs in muscle function has been previously studied. Singh et al.^19^ found that activated APNrs could mitigate dexamethasone‐induced muscle atrophy *in vivo* through the AMPK pathway, thereby simulating PPAR‐γ coactivator 1α. Balasubramanian et al.^52^ observed that APNr activation promoted skeletal muscle repair in aged mice, which was linked to changes in fibre types, especially oxidative fibres. Given that APNr activation could promote osteogenesis and attenuate adipogenesis in AMUSCs, we verified that whether age‐induced skeletal muscle dysfunction could be improved via enhanced myogenesis and decreased adipogenesis under APNr activation. As expected, significant increases in MW/BW, CSA and Feret's diameter were observed after AdipoRon administration. The gene and protein expression levels were consistent with the tissue staining results. Furthermore, the levels of marker factors of muscle senescence were significantly decreased.

Although we have provided promising evidence that APNr activation could affect MUSC function and rescue age‐related skeletal muscle dysfunction, further in‐depth research is required. Owing to the different cellular and internal environments between young and aged states,^53^ the influence of certain senescence‐associated factors on the regulatory effect of APNr activation has not yet been clarified. For the *in vivo* studies, whether AdipoRon administration increases the change in adiponectin secretion should also be explored. The complex endocrine and paracrine systems must also be considered. Furthermore, the results we acquired were from the gastrocnemius in male mice, and additional studies may be extended to the muscles of other body parts and female mice to verify the generality of APNr activation.

## 5 CONCLUSIONS

Overall, our study demonstrated that APNr activation had different effects on myogenic and adipogenic differentiation in aged and YMUSCs. In addition, APNr activation could promote the myogenic differentiation of AMUSCs and inhibit their adipogenic differentiation, further rescuing skeletal muscle atrophy, suppressing fat infiltration and alleviating age‐related skeletal muscle dysfunction.

## CONFLICT OF INTEREST

The author declares that there is no conflict of interest that could be perceived as prejudicing the impartiality of the research reported.

## Supporting information


**FIGURE S1.** Representative haematoxylin and eosin (HE) staining of inguinal fat pads from young and aged mice Bar =100 μm. *n* = 10 male C57BL/6 mice/group.

**FIGURE S2.** qRT‐PCR and western blot results of 17‐day adipogenic differentiation in C2C12 cells (A), young muscle satellite cells (B) and aged muscle satellite cells (C) *p < 0.05, compared with the young group. Data are presented as the mean ± SD from at least three independent experiments.

**FIGURE S3.** (A) Changes in body weight after AdipoRon treatment. (B) Representative haematoxylin and eosin (HE) and oil red O staining of inguinal fat pads from aged mice after AdipoRon treatment for 4 weeks. Bar =100 μm. (C) Representative HE staining of the heart, liver and kidney after AdipoRon treatment for 4 weeks. Bar =100 μm. Data are presented as the mean ± SD from at least three independent experiments (*n* = 10 male C57BL/6 mice/group).

**TABLE S1.** Information of primer sequences used in the experiment.

**TABLE S2.** Information of antibodies used in the experiment.

**TABLE S3.** Information of the siRNA sequences used in the experiment.

## Data Availability

All data needed to evaluate the conclusions in the manuscript are present in the main figures and the Supplementary Materials. Additional related data are available upon request from the authors.
