# Supplementary figures and images for: Adiponectin receptors activation performs dual effects on regulating myogenesis and adipogenesis of young and aged muscle satellite cells
Source: Cell Prolif. 2022 Dec 9;56(3):e13370. doi: 10.1111/cpr.13370 (PMC9977665; doi:10.1111/cpr.13370)

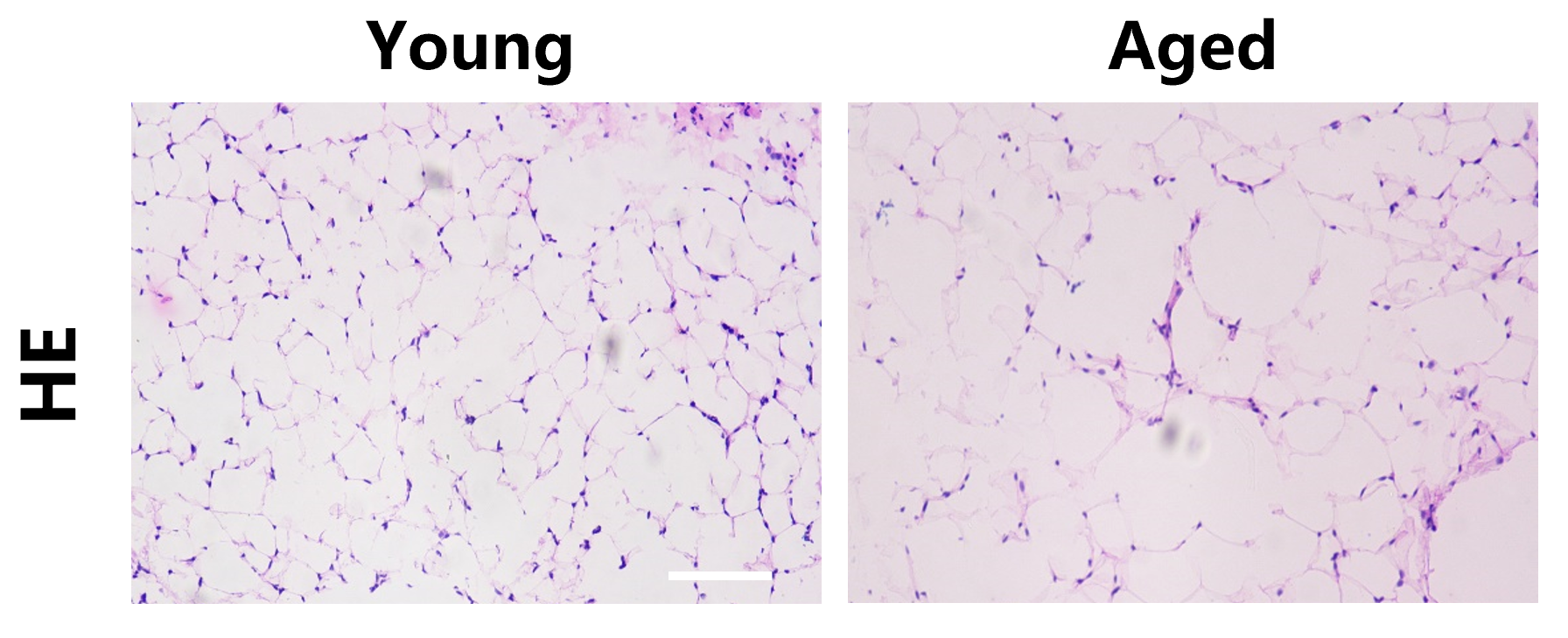

Supplement: Supplementary file 1 — FIGURE S1. Representative haematoxylin and eosin (HE) staining of inguinal fat pads from young and aged mice Bar =100 μm. n = 10 male C57BL/6 mice/group. [file CPR-56-e13370-s003.tif]

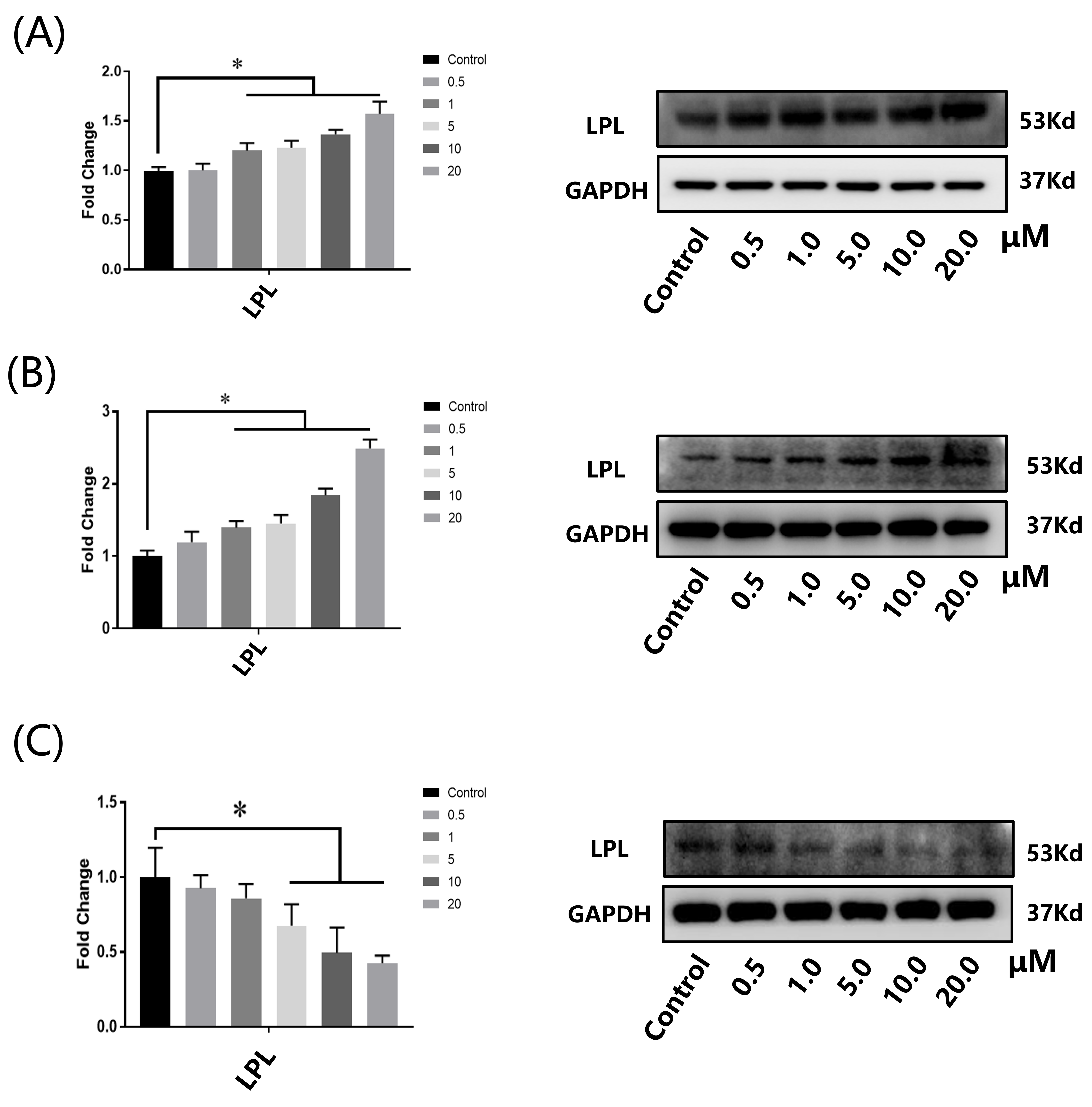

Supplement: Supplementary file 2 — FIGURE S2. qRT‐PCR and western blot results of 17‐day adipogenic differentiation in C2C12 cells (A), young muscle satellite cells (B) and aged muscle satellite cells (C) *p < 0.05, compared with the young group. Data are presented as the mean ± SD from at least three independent experiments. [file CPR-56-e13370-s001.tif]

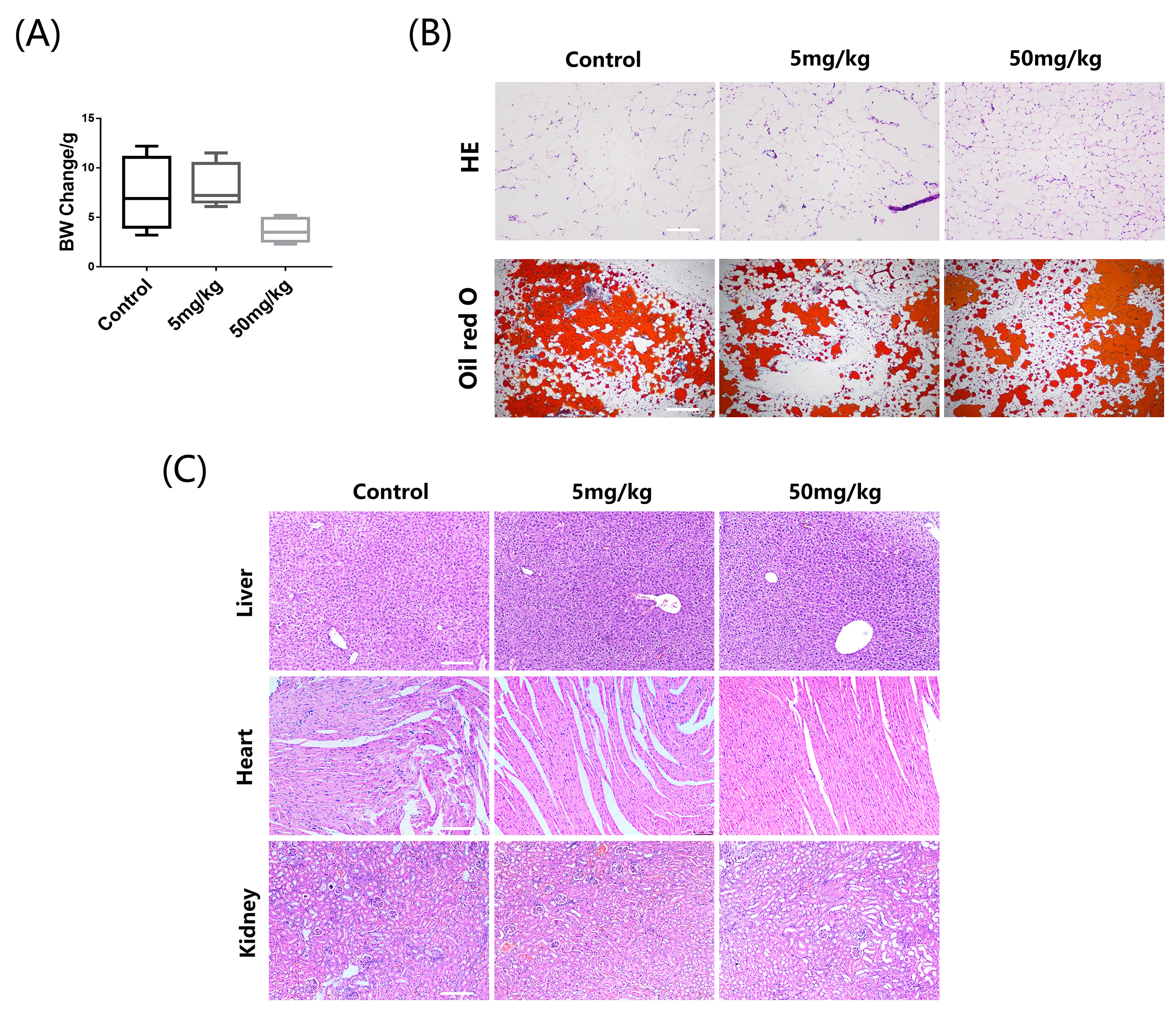

Supplement: Supplementary file 3 — FIGURE S3. (A) Changes in body weight after AdipoRon treatment. (B) Representative haematoxylin and eosin (HE) and oil red O staining of inguinal fat pads from aged mice after AdipoRon treatment for 4 weeks. Bar =100 μm. (C) Representative HE staining of the heart, liver and kidney after AdipoRon treatment for 4 weeks. Bar =100 μm. Data are presented as the mean ± SD from at least three independent experiments (n = 10 male C57BL/6 mice/group). [file CPR-56-e13370-s006.tif]
