# Supplementary material for: Adiponectin receptors activation performs dual effects on regulating myogenesis and adipogenesis of young and aged muscle satellite cells
Source: Cell Prolif. 2022 Dec 9;56(3):e13370. doi: 10.1111/cpr.13370 (PMC9977665; doi:10.1111/cpr.13370)
Supplement: Supplementary file 4 — TABLE S1. Information of primer sequences used in the experiment. [file CPR-56-e13370-s004.docx]

Supplementary Table 1: Information of primer sequences used in the experiment

| Gene | Forward Primer (5’-3’) | Reverse Primer (5’-3’) |
| --- | --- | --- |
| GAPDH | AGGTCGGTGTGAACGGATTTG | TGTAGACCATGTAGTTGAGGTCA |
| Antrogin-1 | CAGCTTCGTGAGCGACCTC | GGCAGTCGAGAAGTCCAGTC |
| MuRF-1 | GTGTGAGGTGCCTACTTGCTC | GCTCAGTCTTCTGTCCTTGGA |
| MyoD | GGATGGTGTCCCTGGTTCTTC | GACTATGTCCTTTCTTTGGGGCT |
| MyoG | AATGCACTGGAGTTCGGTCC | AGTTGGGCATGGTTTCGTCT |
| CEBP-α | CAAGAACAGCAACGAGTACCG | GTCACTGGTCAACTCCAGCAC |
| LPL | GGGAGTTTGGCTCCAGAGTTT | TGTGTCTTCAGGGGTCCTTAG |
| Mck | CTGACCCCTGACCTCTACAAT | CATGGCGGTCCTGGATGAT |
| Myosin | AAGTGACTGTGAAAACAGAAGCA | GCAGCCATTTGTAAGGGTTGAC |
| PPAR-γ | GGAAGACCACTCGCATTCCTT | GTAATCAGCAACCATTGGGTCA |
