# Supplementary material for: Adiponectin receptors activation performs dual effects on regulating myogenesis and adipogenesis of young and aged muscle satellite cells
Source: Cell Prolif. 2022 Dec 9;56(3):e13370. doi: 10.1111/cpr.13370 (PMC9977665; doi:10.1111/cpr.13370)
Supplement: Supplementary file 5 — TABLE S2. Information of antibodies used in the experiment. [file CPR-56-e13370-s005.docx]

Supplementary Table 2: Information of antibodies used in the experiment

| Antibody | Brand | Product code (#) | Dilution concentration |
| --- | --- | --- | --- |
| GAPDH | Signalway Antibody | 41549 | WB 1:3000 |
| Antrogin-1 | HUABIO | ET7109-25 | WB 1:500; IF 1:100 |
| MuRF-1 | Proteintech | 55456-1-AP | WB 1:1000; IF 1:50 |
| MyoD | bimake | A5766 | WB 1:500 |
| CEBP-α | (HUABIO | ET1612-46 | WB 1:500 |
| LPL | abcam | ab21356 | WB 1:1000 |
| AMPK | Cell Signaling | 2532 | WB 1:1000 |
| P-AMPK(Thr172) | Cell Signaling | 50081 | WB 1:1000 |
| PPAR-α | abcam | ab126285 | WB 1:1000 |
| myosin | R&D | MAB4470 | WB 1:500; IF 1:25 |
| MyoG | abcam | ab124800 | WB 1:200 |
| PPAR-γ | HUABIO | ET1702-57 | WB 1:500 |
| AdipoR1 | HUABIO | ET1610-86 | WB 1:500; IF 1:100 |
| AdipoR2 | Bioss | bs-0611R | WB 1:1000; IF 1:100 |
| GSK-3β | HUABIO | ET1607-71 | WB 1:1000 |
| P-GSK-3β | HUABIO | ET1607-60 | WB 1:1000 |
| β-catenin | HUABIO | EM0306 | WB 1:1000 |
| P-β-catenin | Cell Signaling | 4167 | WB 1:1000 |
| c-Myc | Proteintech | 10828-1-AP | WB 1:2000 |
| CyclinD1 | Proteintech | 26939-1-AP | WB 1:2000 |
| PI3K | Cell Signaling | 4292 | WB 1:1000 |
| P-PI3K | Cell Signaling | 4228 | WB 1:1000 |
| Akt | Cell Signaling | 4691 | WB 1:1000 |
| P-AKT | Cell Signaling | 4060 | WB 1:1000 |
| HRP Conjugated Goat anti-Rabbit IgG Goat Polyclonal Antibody | HUABIO | HA1001 | WB 1:10000 |
| FITC Conjugated Goat anti-Rabbit IgG Goat Polyclonal Antibody | HUABIO | HA1004 | IF 1:300 |
| TRITC Conjugated Goat Anti-Rabbit IgG H&L Goat Polyclonal Antibody | HUABIO | HA1016 | IF 1:200 |
| Note: WB: Western Blot IF: Immunofluorescence | | | |
