# Supplementary material for: Adiponectin receptors activation performs dual effects on regulating myogenesis and adipogenesis of young and aged muscle satellite cells
Source: Cell Prolif. 2022 Dec 9;56(3):e13370. doi: 10.1111/cpr.13370 (PMC9977665; doi:10.1111/cpr.13370)
Supplement: Supplementary file 6 — TABLE S3. Information of the siRNA sequences used in the experiment. [file CPR-56-e13370-s002.docx]

Supplementary Table 3: Information of the siRNA sequences used in the experiment

| siRNA | Sense (5’-3’) | Antisense (5’-3’) |
| --- | --- | --- |
| scramble siRNA | UUCUCCGAACGUGUCACGUTT | ACGUGACACGUUCGGAGAATT |
| AdipoR1 siRNA | GGCUCUAUUACUCCUUCUATT | UAGAAGGAGUAAUAGAGCCTT |
| AdipoR2 siRNA | CGGAUUGGCUUAAGGAUAATT | UUAUCCUUAAGCCAAUCCGTT |
